# Safety and efficacy of a mesenchymal stem cell intramammary therapy in dairy cows with experimentally induced *Staphylococcus aureus* clinical mastitis

**DOI:** 10.1038/s41598-020-59724-7

**Published:** 2020-02-18

**Authors:** O. A. Peralta, C. Carrasco, C. Vieytes, M. J. Tamayo, I. Muñoz, S. Sepulveda, T. Tadich, M. Duchens, P. Melendez, A. Mella, C. G. Torres

**Affiliations:** 1 Department of Animal Production Science, Faculty of Animal and Veterinary Sciences, University of Chile, Santiago, 8820808 Chile; 2 Department of Biomedical Sciences and Pathobiology, Virginia-Maryland Regional College of Veterinary Medicine, Virginia Tech, Blacksburg, VA 24060 USA; 3 Food Animal Health & Management Program, College of Veterinary Medicine, University of Georgia, Athens, GA 30602 USA; 4 Mastitis Laboratory, Department of Biochemistry and Microbiology, Faculty of Sciences, Austral University of Chile, Valdivia, 5110566 Chile; 5 Department of Clinical Sciences, Faculty of Animal and Veterinary Sciences, University of Chile, Santiago, 8820808 Chile

**Keywords:** Animal biotechnology, Stem-cell biotechnology, Bacterial infection

## Abstract

Although, antibiotics are effective in the treatment of bovine mastitis, they do not address the regeneration of mammary glandular tissue and have been associated to the increment in antimicrobial resistance worldwide. Considering the necessity of alternative therapies for this disease of high economic impact and the reported regenerative and antibacterial effects of mesenchymal stem cell (MSCs), we evaluated the safety and efficacy of an allogenic MSC-based intramammary therapy in dairy cows with experimentally induced *Staphylococcus aureus* clinical mastitis. In a safety trial, heifers were inoculated intramammarily with a 2.5 × 10^7^-suspension of bovine fetal AT-MSCs on experimental days 1 and 10. Animals were evaluated clinically on a daily basis during a 20-day experimental period and blood samples were collected for hemogram determination and peripheral blood leukocytes (PBLs) isolation. In an efficacy trial, Holstein Friesian cows were inoculated with *S. aureus* and treated intramammarily with vehicle (NEG; days 4 and 10), antibiotics (ATB; days 4 and 5) or a suspension of 2.5 × 10^7^ AT-MSCs (MSC; days 4 and 5). Cows were clinically evaluated daily and milk samples were collected for somatic cell count (SCC) and colony forming units (CFU). Blood samples were collected for serum haptoglobin and amyloid A determination. Intramammary administration of two doses of bovine fetal AT-MSCs in healthy cows did not induce changes in clinical or hematological variables, and gene expression profiles in PBLs associated to activation (CD4, CD8, CD25, CD62L and CD69) and proinflammatory cytokines (CCL2, CCL5, IL2, CXCL3, IFNγ, and TNFα). Quarters of MSC group of cows had similar SCC log/mL in milk compared to infected quarters of ATB or NEG cows. However, quarters of MSC cows had lower CFU log/mL in milk compared to quarters of NEG cows. Intramammarily inoculation of repeated doses of 2.5 × 10^7^ allogenic AT-MSCs did not induce clinical or immunological response in healthy cows. Moreover, MSC-intramammary treatment reduced bacterial count in milk of cows with *S. aureus* clinical mastitis compared to untreated cows. This work provides initial evidence for the safety and efficacy of an allogenic MSC-based intramammary therapy for the treatment of bovine mastitis.

## Introduction

Currently, the treatment of bovine mastitis is based on the use of antibiotics, which seeks to control the growth of bacteria responsible for the infection of the mammary gland. Although, antibiotic therapy is the most efficient treatment to control mastitis, they do not address the regeneration of mammary glandular tissue, whose integrity is fundamental in terms of milk production. Moreover, antibiotic use in dairy cows is under continue scrutinization due to eventual association to the increment in antimicrobial resistance and the presence of antibiotic residues in milk. Considering that the development of alternative or complementary therapies for the treatment of bovine mastitis is imperative, the recent scientific advances in the area of regenerative medicine represent a potential tool that deserves to be evaluated.

*Staphylococcus aureus* is a contagious pathogen involved in persistent intramammary infection with a prevalence in heifers that range from 0 to 15%^1^. This pathogen is part of the natural skin flora, making its eradication from herds impossible. Some of the mechanisms of virulence include adhesion to mammary epithelial cells and production of exotoxins, biofilms, bacterial super-antigens, and proteases^2^. In addition, some strains are capable of suppressing phagocytosis and cellular immunity, producing an enzyme that inactivates the vast majority of penicillin-based treatments^3^. Moreover, *S. aureus* can survive inside the epithelial cells and phagocytes, so few antimicrobial agents are able to reach the entire affected area^4^. Cure rates of most therapies against *S. aureus* is around 50% with reinfections usually due to primary infectious organism rather than to new pathogens^5^. Consequently, antibiotic therapy against *S. aureus* is often ineffective and results in chronic udder infections^6^. Thus, development of alternative or complementary treatments for *S. aureus* intramammary infection is required in order to reduce bacterial resistance and increase cure rates in lactating dairy cows.

Regenerative medicine is a growing area that uses stem cell populations or their secreted products to treat a variety of disease states in both humans and animals. Mesenchymal stem cells (MSCs) are multipotent progenitor cells located in several tissues including adipose (AT-MSCs). Evidence indicates that MSCs correspond to perivascular cells known as pericytes with the capacity to detach from vessels when tissues are damaged or inflamed^7^. Although MSCs have capacity for differentiation into medodermal lineages including osteogenic, chondrogenic and adipogenic, their therapeutic potential is based on production of immunomodulatory, angiogenic and antibacterial factors^8^. We have recently reported that stimulation of bovine fetal AT-MSCs with inflammatory interferon γ (IFNγ) induce up-regulation of immunomodulatory factors indolamine 2,3-dioxygenase (IDO) and interleukin 6 (IL-6)^9^. In addition, bovine fetal AT-MSCs are able to induce angiogenesis under *in vitro* conditions, which may be mediated by expression of vascular endothelial growth factor (VEGF) and angiopoietin 1 (ANGPT1)^10^. Moreover, conditioned media from bovine fetal AT-MSCs induce an anti-proliferative effect against mastitis-causing *S. aureus*, which may be mediated by antibacterial peptides (AP) defensin β1 (DEFβ1) and NK Lysin^11^. These studies suggest that bovine fetal AT-MSCs are able to produce several factors that may be useful to control a variety of infectious conditions including bovine mastitis.

The potential use of MSCs in cell therapy is also based on their reduced immunogenicity known as the immune evasive potential that enable MSC allogenic transplantation^12^. Reduced expression of major histocompatibility complexes I and II (MHC-I and II), and lack of T-cell costimulatory molecules CD80 suggest that bovine fetal AT-MSCs might display immune evasive potential as well^9^. Reduced immunogenicity, together with their immune regulatory abilities aforementioned, would allow bovine fetal AT-MSCs to evade recognition by immune system in recipients, and thus may be used as an allogenic therapeutic strategy. Thus, the aim of the present study was to evaluate the safety of an MSC intramammary therapy in healthy cows and its efficacy in dairy cows with experimentally induced *S. aureus* clinical mastitis.

## Material and Methods

### Bovine fetal AT-MSC isolation and culture

AT-MSCs were harvested from fetal AT present in the abdominal omentum from male bovine fetuses (n = 9; 7–8 months of gestation) collected at a local abattoir^9–11^. Approximately 10 g of AT were isolated under aseptic conditions and deposited in PBS supplemented with 100 IU/mL penicillin, 100 μg/mL streptomycin and 2.5 μg/mL amphotericin B (Hyclone, Thermo Fisher Scientific, UT, USA). AT samples from three fetuses were pooled and washed twice with phosphate-buffered saline (PBS; Hyclone) and twice with high glucose (4.5 g/L) Dulbecco’s Modified Eagle Medium (DMEM, Gibco, Grand Islands, NY, USA) supplemented with 100 IU/mL penicillin, 100 μg/mL streptomycin and 2.5 μg/mL amphotericin B (Hyclone,). Then, AT was digested in 0.5% collagenase I (Sigma-Aldrich, St. Louis MO, USA) (1 ml/g of AT) for 45 min. Collagenase I activity was neutralized with DMEM supplemented with 10% fetal bovine serum (FBS; Gibco), 100 IU/mL penicillin, 100 μg/mL streptomycin and 2.5 μg/mL amphotericin B (expansion medium). The disrupted tissue was filtered through 40 μm pores and subsequently centrifuged at 400 × g for 5 min. The cell pellet was washed once in DMEM, suspended in expansion medium and plated. The sediment was resuspended in expansion medium, then transferred to 175 cm^2^ cell culture flasks. The cultures of MSCs derived from adipose tissue were incubated at 38 °C under a humid atmosphere with 5% CO_2_. After 48 hours, the cells not adherent to the plastic were removed by means of a change of culture medium. Upon reaching 80 to 90% confluence, AT-MSC passages were made by removal with trypsin/EDTA, supplemented with 250 μg/mL of amphoterecin B, 100 IU/mL of penicillin and 100 μg/mL of streptomycin.

### Safety study design

**Figure 1:**
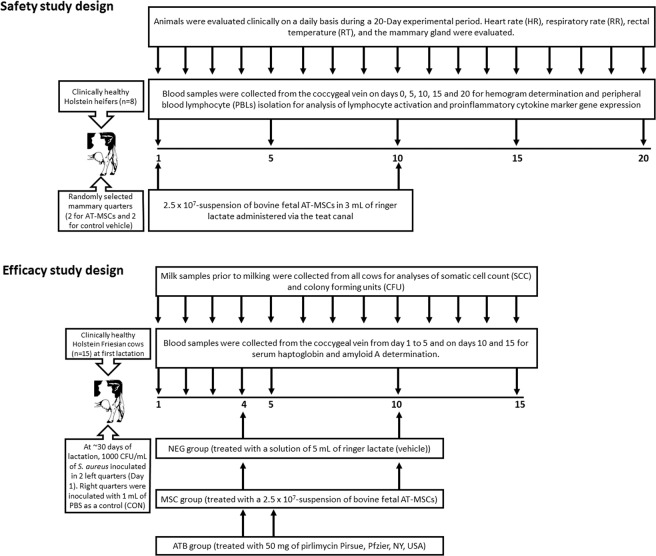
Experimental designs for safety and efficacy studies of an allogenic MSC-based intramammary therapy in dairy cows with experimentally induced *Staphylococcus aureus* clinical mastitis.

All experimental procedures have been approved and were performed in accordance with guidelines and regulations of the Bioethical Committees of the Scientific and Technological Development Support Fund from Chile (Fondef) and the Faculty of Animal and Veterinary Sciences at University of Chile (Certificate N° 04-2015). Clinically healthy Holstein heifers (n = 8) of approximately 15 months of age, weighing an average of 445 kg, belonging to a commercial dairy located in the Valparaíso Region of Chile, were used in the safety trial (Fig. 1). Heifers were managed under confinement and fed a total mixed ration based on silage of corn, alfalfa hay and concentrate. On experimental days 1 and 10, a 2.5 × 10^7^-suspension of bovine fetal AT-MSCs in 3 mL of ringer lactate was administered via the teat canal in two randomly selected mammary quarters. In the remaining two mammary quarters, a 3 mL solution of ringer lactate was administered as control (vehicle). Animals were evaluated clinically on a daily basis during a 20-Day experimental period. Heart rate (HR), respiratory rate (RR), and rectal temperature (RT) were determined. The clinical evaluation of the mammary gland was based on a previously published method that includes the observation of volume increase, firmness, signs of pain and milk secretion^13^. In addition, the mammary surface temperature (MST) of each quarter was quantified using a portable thermograph (Fluke VT02 Washington, USA). Mammary temperature difference (MTD) was calculated by subtracting MST from quarters inoculated with AT-MSCs from quarters inoculated with vehicle. Blood samples were collected from the coccygeal vein on days 0, 5, 10, 15 and 20 for hemogram determination and peripheral blood leukocytes (PBLs) isolation. Hemogram was analyzed in a commercial laboratory (LQCE, Santiago, Chile).

### Isolation of peripheral blood leukocytes

Blood samples were transferred to the laboratory and the blood was mixed with PBS (Corning, NY, USA) in equal parts (1:1). Subsequently, the same volume of histopaque (Sigma-Aldrich, Saint Louis, MO, USA) was added at room temperature and blood mixed with PBS was added slowly and unmixed over this solution^14^. Subsequently, the solution was centrifuged at 900 × g for 35 min, to achieve a separation in four phases: (1) plasma with PBS, (2) peripheral blood mononuclear cells, (3) histopaque solution and (4) granulocytes and red blood cells. The PBLs was extracted, mixed with PBS and centrifuged at 1100 × g for 10 min. The pellet was resuspended in PBS and centrifuged at 800 × g for 5 min. Finally, the new pellet was resuspended in RPMI (Biological Industries, Beit Haemek, Israel) supplemented with 10% fetal bovine serum (Gibco, Waltham, MA, USA) and 250 μg/mL of amphoterecin B, 100 IU/mL of penicillin and 100 μg/mL of streptomycin (Corning). Subsequently, the PBL pellet was seeded in petri dishes with RPMI medium and incubated at 38.5 °C with 5% CO_2_ for two hours. To conserve the PBLs, the non-adherent cells were removed from the plastic and centrifuged at 800 × g for 5 min. The cell pellet was fixed in lysis buffer (Thermo Scientific, Waltham, MA, USA) with 2β-Mercaptoethanol (1:50, Sigma-Aldrich) and were frozen at −20 °C until further analysis.

### RNA extraction, cDNA synthesis and quantitative PCR

Purification of total RNA from PBLs was performed using a GeneJET purification kit (Thermo Scientific, Vilnus, Lithuania), following the manufacturer’s instructions^9–11^. Total RNA was extracted using GeneJET RNA purification kit (Thermo Scientific) according to the manufacturer’s instructions. Total RNA was eluted in 50 mL of RNase free water. The concentration and purity of the RNA in each sample was determined using Qubit RNA assay kit (Life Technologies, Waltham, MA, USA), and genomic DNA was removed using RNase-free DNase I (Thermo Scientific). Samples were subjected to complementary DNA (cDNA) synthesis using a Brilliant II SYBR Green QRT-PCR AffinityScript Master Mix, 2-step kit (Agilent Technologies, Santa Clara, CA, USA). The reaction protocol consisted of incubation for 5 min at 25 °C, 15 min at 42 °C, 5 min at 95 °C, and hold at 4 °C using a TC1000-G gradient thermocycler (SciLogex, Rocky Hill, CT, USA). Samples were analyzed for expression of lymphocyte activation marker genes CD4, CD8, CD25, CD62L and CD69, proinflammatory cytokine marker genes IL2, IFNγ, TNFα, CCL2, CCL5 and CXCL3, and endogenous genes βACTIN and GAPDH^15^ by quantitative-PCR (Q-PCR) (Table 1). Primers were designed using the National Center for Biotechnology Information (NCBI) database to find the required nucleotide sequences (Table 1). Subsequently, the NCBI primer design tool was used and the performance of the primers for (Q-PCR) was verified using the Premier Biosoft particle analysis software. Equivalence of amplification efficiencies among all primer-probe sets was confirmed using serial 3-fold dilutions of PBL cDNA. Each PCR reaction (10 µL) contained the following: 2X Brilliant II SYBR Green QPCR master mix (5 µL), target forward primer (200 nM), target reverse primer (200 nM), cDNA synthesis reaction (1 µL), and nuclease-free PCR grade water to adjust final volume. The PCR amplification was carried out in an Eco Real-Time PCR System (Illumina Incorporated, San Diego, CA, USA). Thermal cycling conditions were 95 °C for 10 min, followed by 40 repetitive cycles at 95 °C for 30 s, and 60 °C for 1 min. Amplifications were followed by dissociation (melting) curves to ensure specificity of the primers. In each experiment, amount of gene expression was recorded as CT values that corresponded to the number of cycles where the fluorescence signal can be detected above a threshold value. The CT averages for each biological replicate were calculated and transformed into relative values denominated quantity (Q) through ∆∆CT formula^16^. Then, the relative quantification in the expression of target genes for each sample was estimated as the quotient between Q value of the target gene and a normalization factor (NF), which was calculated based on the geometric mean of housekeeping gene Q values.

**Table 1:** Sequence of primers used for Q-PCR analysis.

Gene	Nucleotide sequence (5′-3′)	Accession number
**Endogenous genes**		
β- ACTIN	Forward CGCACCACTGGCATTGTCAT Reverse TCCAAGGCGGACGTAGCAGAG	NM_173979.3
GAPDH	Forward CCTTCATTGACCTTCACTACATGGTCTA Reverse CCTTCATTGACCTTCACTACATGGTCTA	NM_001034034.2
**Lymphocyte activation genes**		
CD4	Forward TTCCTTCCCACTCACCTTCG Reverse ATCTTGTTCACCTTCACCTCTC	XM_024991418.1
CD8	Forward AAGGCATACCACAAGGGTTATC Reverse CACAGCCAGGTTCTGAGAATAG	NM_174015.1
CD25	Forward GGAAAGCCCTAACACTGATGTA Reverse AGAGGCTTGGAAAGGACTTATG	NM_174358.2
CD62L	Forward TACGAAAGGACGGAGCAAAG Reverse GAGCATAATCCAGACCCACAG	NM_174182.1
CD69	Forward GGGTCCATTCAAGTTCCTATCC Reverse TGTACTGGCCCACTGATAGA	NM_174014.2
**Proinflammatory cytokine genes**		
IL2	Forward GGGAACACAATGAAAGAAGTGAAG Reverse GAGCTTGAGGTTCTCAGGATTT	NM_180997.2
IFNɣ	Forward AACACAGGAGCTACCGATTTC Reverse AAGCCCACAGAGCAGTAAAG	NM_174086.1
TNFɑ	Forward GCCAACTCCCTCTGTTTATGT Reverse GACACCTTGACCTCCTGAATAA	NM_173966.3
CCL2	Forward TCGCCTGCTGCTATACATTC Reverse CACAGCCTCTTTAGGACACTT	NM_174006.2
CCL5	Forward CCTGCTGCTTTGCCTATATCT	NM_175827.2
	Reverse GCGCTTCTTCCTGGTGATAA	
CXCL3	Forward ACATAACCCAGTCCTGATTGTT	NM_001046513.2
	Reverse CACAGAGCCTGGCACTTTAT	

### Efficacy study design

The efficacy trial was performed in facilities of the Faculty of Animal and Veterinary Sciences at University of Chile. Clinically healthy Holstein Friesian cows (n = 15) at first lactation were used in the study (Fig. 1). Animals were fed alfalfa hay and concentrate. Cows were milked twice a day using a separate milking unit and the milk production was quantified after each milking. Only cows with three consecutive negative cultures were used in the study. At 29.8 ± 7.1 days of lactation, cows were inoculated in the two left quarters with a suspension of *S. aureus* and were randomly assigned to three experimental groups of 5 animals each. The NEG group (without therapy) was treated intramammarily in the two infected mammary quarters with an solution of 5 mL of ringer lactate (vehicle) on experimental days 4 and 10. The ATB group (antibiotic therapy) was treated intramammarily in the two infected mammary quarters with a commercial suspension of 50 mg of pirlimycin (Pirsue, Pfzier, NY, USA) on experimental days 4 and 5. The MSC group (therapy with AT-MSCs) was treated intramammarily in the two infected mammary quarters with a suspension of 2.5 × 10^7^ of MSCs in 3 mL of ringer lactate on experimental days 4 and 10. Right quarters were inoculated at day 1 with 1 mL of PBS as a control (CON). From the day of challenge and daily for 15 days, cows were evaluated by clinical examination that included determination of HR, RR, RT and MST. The clinical evaluation of the mammary gland was performed as described above. Abnormalities in the appearance of the milk were evaluated at the beginning of milking using a strip cup with dark surface to classify the secretion as normal, aqueous, viscous, with the presence of flakes or lumps, blood or pus. Milk samples prior to milking were aseptically collected for somatic cell count (SCC) and colony forming units (CFU) from all cows following the protocols of the International Dairy Federation^17^ from three consecutive days prior to the experimental challenge and daily during the 15 days of the study. A cow was considered to be cured when all infected and noninfected quarters at pre-treatment samplings were negative for *S. aureus* at the 3 post-treatment daily cultures. Blood samples (10 ml) were collected from the coccygeal vein from day 1 to 5 and on days 10 and 15 into vacutainers for serum haptoglobin and amyloid A determination.

### Preparation of *S. aureus* inoculum

Three bacterial cultures of milk were performed to rule out the presence of intramammary infections. The strain of *S. aureus* UACH-70^18^ was kindly provided by Dr. Armin Mella from the Institute of Biochemistry and Microbiology of the Faculty of Sciences of the Austral University of Chile. Once thawed, the strain was seeded on blood agar and incubated overnight at 37 °C in aerobiosis. A bacterial colony was then incubated in 5 mL of brain heart broth in a 15 mL tube for 10 hours at 37 °C. Subsequently, eight dilutions of the broth were prepared in PBS and 100 μL of blood agar was seeded in duplicate from each dilution. Using a sterile rake, the inoculum of each dilution was distributed on the surface of each plate. The plates were incubated overnight in aerobiosis at 37 °C. Subsequently, duplicate plate colonies containing between 30–300 CFU were counted and averages were obtained. To calculate the number of CFU/mL, the following formula was used: Number of colonies × (10)^(number of dilution + 1)^ = CFU/mL. The volume needed to obtain a bacterial suspension with a concentration of 1000 CFU/mL in 1 lt of PBS was then calculated. Cows approximately 30 days postpartum, were challenged by intracisternal inoculation of two left mammary quarters using 1 mL of a suspension of 1000 CFU/mL of *S aureus* UACH-70. Right quarters were inoculated with 1 mL of PBS as a control (CON).

### Somatic cell count and colony forming unit determination

Milk samples were collected from all the cows in 50 mL plastic tubes with bronopol as a preservative and refrigerated at 4 °C. The samples were sent under refrigeration to a commercial laboratory (Labsur, Osorno, Chile) for determination of SCC. The cell count was performed using a Fossomatic flow cytometer (FC 5000, Foss, Denmark). Additional milk samples were collected in sterile 15 mL tubes and frozen at −20 °C. Bacteriological analyzes were performed according to accepted standards and recommended by the National Mastitis Council, USA^19^. An aliquot of 0.01 mL of each fresh milk sample was grown on blood agar plates (Oxoid Diagnostic Reagents, UK) supplemented with 5% sheep blood. The presumptive colonies of *S. aureus* based on their morphology were subcultured in a new blood agar plate and tested for coagulase production by means of a test tube with rabbit plasma with EDTA (Bactident Coagulase, Merck, Germany). For the identification of *S aureus* was used in rapid kit Staphytect Plus (Oxoid Diagnostic reagents, UK).

### Haptoglobin and amyloid A determination

Blood was allowed to coagulate at room temperature for 1 h and centrifuged at 1000 × g for 20 min at 4 °C, and serum was stored at −20 °C until assay. The levels of haptoglobin in serum were measured using an ELISA kit (Life Diagnostics, Inc., West Chester, PA, USA) according to the manufacturer’s instructions. The amounts of serum amyloid A were determined using a semi-quantitative test (EquiCheck from Accuplex Diagnostics, Maynooth, Ireland) according to the manufacturer’s instructions.

### Analysis of data

The normality of the data on clinical and hemogram variables, milk yield, gene expression, and haptoglobin and amyloid A levels was evaluated using the Shapiro Wilks normality test. Accordingly, SCC and CFU values were not normally distributed and were log-transformed. For the safety trial, clinical, hemogram and gene expression values were evaluated as dependent variables and the day of sampling was evaluated as independent variable using repeated measures ANOVA. For the efficiency trial, clinical, milk yield, haptoglobin and amyloid A values were evaluated as dependent variables and the day of sampling and treatments groups were evaluated as independent variable using repeated measures ANOVA. The mean of values between days of sampling and treatments groups were analyzed using Duncan’s multiple comparison test (P < 0.05). For the efficacy trial, the SCC and CFU values were compared between groups using a linear mixed model with treatment, day of sampling and mammary quarters as fixed effects and cow as random effects. Statistical analyzes were performed using the software Info Stat (Córdoba, Argentina, 2008).

### Ethics approval and consent to participate

All procedures have been approved by the Bioethical Committees of the Scientific and Technological Development Support Fund (FONDEF) Grant ID15I10129, Government of Chile and the University of Chile.

### Consent for publication

All authors critically revised the manuscript for important intellectual contents and approved the final manuscript.

## Results

### Intramammary administration of bovine fetal AT-MSCs in healthy heifers did not induce clinical effect, immune rejection or memory

**Figure 2:**
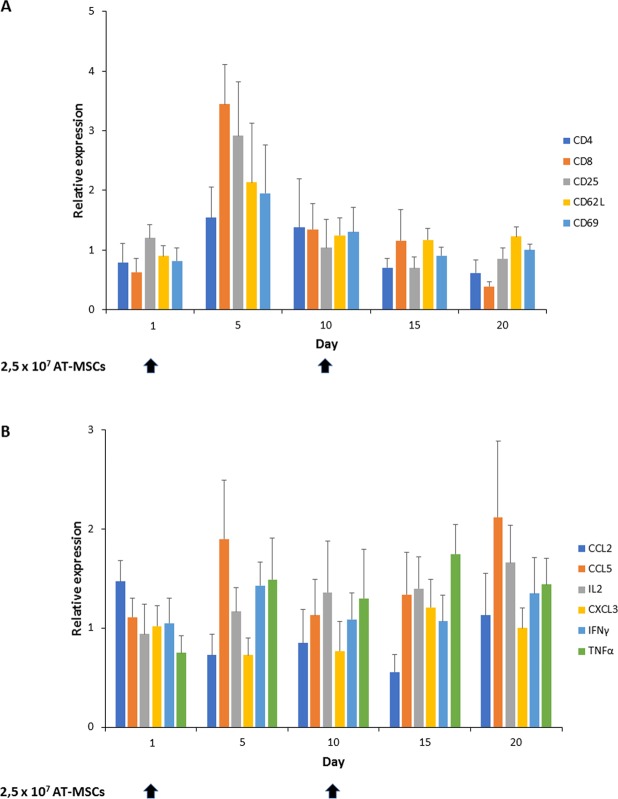
Gene expression profiles in peripheral blood leukocytes (PBLs) associated to activation and proinflammatory cytokines in healthy dairy heifers treated with two doses of allogenic bovine fetal AT-MSCs. No significant differences (P > 0.05) were found in gene expression profiles in PBLs associated to activation (**A**) and proinflammatory cytokines (**B**) in blood samples collected from healthy dairy heifers (n = 8) every five days during a 20-Day period. Black arrows represent AT-MSCs intramammary inoculation at days 1 and 10.

In order to determine the potential immunogenic effect of an allogenic bovine fetal AT-MSC intramammary therapy in dairy cows; clinical and hematological variables in cows, and gene expression profiles in PBLs were evaluated every five days during a 20-day experimental period. Healthy dairy heifers were treated intramammarily with two doses of AT-MSCs separated by 10 days (Days 1 and 10) with the aim to evaluate if MSCs were able to generate immune rejection and memory. Clinical variables including RR, HR, and RT were within reference intervals and were not affected (P > 0.05) by intramammary inoculation of AT-MSCs (Table 2). Moreover, MTD in quarters were not different (P > 0.05) during the experimental period. Hematological variables including erythrocytes, neutrophils, lymphocytes, eosinophils, monocytes, and platelets counts, and hemoglobin, PCV, fibrinogen and globulin levels were within reference intervals and were not different (P > 0.05) between sampling days. In addition, gene expression profiles of lymphocyte activation markers CD4, CD8, CD25, CD62L and CD69 and of proinflammatory cytokine markers CCL2, CCL5, IL2, CXCL3, IFNγ, and TNFα in PBLs were not significantly different between sampling days (Fig. 2).

**Table 2:** Clinical and hematological values in healthy dairy cows treated with an intramammary suspension of AT-MSCs.

Item^a^	Reference^b^	Days					
		0^c^	5	10^c^	15	20	P
RR (bpm)	12–36	54.7 ± 3.7	55.5 ± 4.4	60.7 ± 8.8	54.0 ± 16	51.3 ± 5.5	0.6
HR (bpm)	40–80	84.0 ± 3.1	80.3 ± 7.2	82.3 ± 7.1	71.7 ± 5.8	74.0 ± 2.0	0.15
RT (°C)	38–39	38.9 ± 0.1	39.2 ± 0.1	39.3 ± 0.2	38.5 ± 0.4	38.9 ± 0.3	0.13
MTD (°C)		0.8	−1,1	−0.4	0.8	−0.5	0.08
Erythrocytes (1 × 10^6^/L)	5–10	6.2 ± 0.34	6.4 ± 0.12	5.8 ± 0.17	5.8 ± 0.1	6.4 ± 0.17	0.14
Hemoglobin (g/dL)	8–15	10.2 ± 0.5	10.2 ± 0.2	9.2 ± 0.4	10.1 ± 0.1	10.2 ± 0.2	0.12
PCV (%)	24–46	29.1 ± 1.7	29.6 ± 0.5	26.8 ± 1.0	29.1 ± 0.3	29.4 ± 0.7	0.5
Leukocytes (1 × 10^3^/µL)	4–12	6 ± 0.5	7.5 ± 0.3	8.2 ± 0.4	8.3 ± 0.5	8.4 ± 0.6	0.14
Neutrophils (1 × 10^3^/µL)	0.6–4	2.88 ± 0.21	2.77 ± 0.19	2.71 ± 0.22	2.85 ± 0.18	2.89 ± 0.21	0.34
Lymphocytes (1 × 10^3^/µL)	2.5–7.5	4.1 ± 0.2	3.7 ± 0.2	4.5 ± 0.3	4.4 ± 0.2	4.3 ± 0.3	0.4
Eosinophils (1 × 10^3^/µL)	0–2.4	1.46 ± 0.75	1.12 ± 1.7	1.1 ± 0.67	1.18 ± 1.81	1.7 ± 1.36	0.16
Monocytes (1 × 10^3^/µL)	0.025–0.84	0	0	0	0	0	0.40
Platelet count (1 × 10^−3^/L)	100–800	407 ± 16.2	402 ± 10.4	403 ± 10.98	402 ± 10.21	406 ± 8.24	0.29
Fibrinogen (mg/dL)	200–500	369 ± 23.8	265 ± 44.9	330 ± 31.4	248 ± 48.3	306 ± 32.7	0.28
Globulin (g/dL)	2.9–4.9	3.4 ± 0.13	3.5 ± 0.11	3.5 ± 0.11	3.5 ± 0.14	3.4 ± 0.1	0.14

^a^RR = respiratory rate; HR = heart rate; RT = rectal temperature; MTD = mammary temperature difference; PCV = packed cell volume.

^b^Smith *et al*., 2014. ^c^2,5 × 10^7^ AT-MSCs were administered intramammarily on Days 0 and 10.

### Intramammary administration bovine fetal AT-MSCs in cows with mastitis caused by *S. aureus* did not induce changes in clinical variables or haptoglobin and amyloid A serum concentrations

In order to evaluate the therapeutic potential of an allogenic bovine fetal AT-MSCs intramammary treatment in dairy cows with experimentally induced *S. aureus* mastitis; clinical variables, milk yield, haptoglobin and amyloid A in serum, SCC and UFC in milk were evaluated daily during a 15-Day experimental period. RR and HR were among the reference interval considered for adult Holstein cows (RR: 12–36 breaths per min, bpm; HR:40–8 beats per min, bpm, Table 3)^20^ and no significant differences were detected between experimental groups. Mean RT values were among the reference interval considered for adult Holstein cows (38–39 °C)^20^; however, at the end and middle of the trial, mean RT of ATB and MSC groups of cows had values below the reference interval. No significant differences were detected in mean MTD during that experimental period. Average surface temperatures between infected and control quarters were (range, max-min °C) 37.5–30.4 and 36.9–30.4 in NEG cows, 37–29.8 and 36.9–28 in ATB cows, and 36.9–31.8 and 37.2–30.9 in MSC cows. Moreover, difference in average surface temperatures between infected and control quarters were (range, max-min °C) 2.3–0.7 in NEG cows, 0.7–0.2 in ATB cows, and 1.6–0.1 in MSC cows (Table 3).

**Table 3:** Clinical values in dairy cows with *S. aureus*-induced mastitis and treated with an intramammary suspension of AT-MSC.

Item^a^	Treat.	Days post-infection															
		1	2	3	4^a,c^	5^c^	6	7	8	9	10^b^	11	12	13	14	15	P
RR (bpm)	NEG	37.6 ± 3.8	27.5 ± 2.1	31 ± 4.5	27.5 ± 3.8	29 ± 3.1	31 ± 3	28.5 ± 1.5	34.5 ± 3.5	26.5 ± 1.3	31 ± 2.2	31 ± 2.2	30.5 ± 2.6	28 ± 1.2	32 ± 2.4	32 ± 3.2	0.32
	ATB	31.2 ± 4.8	29.2 ± 1.9	29.6 ± 2.7	30 ± 4.8	25.2 ± 2.3	26 ± 2.3	27.4 ± 1.5	30.8 ± 2.5	29.6 ± 1.6	28.8 ± 2.7	28 ± 1.8	29.2 ± 2.2	26.8 ± 2.2	27.3 ± 1.9	30.6 ± 2.7	0.15
	MSC	29.6 ± 2.7	32 ± .2.2	32 ± 2.8	30.4 ± 2.4	28 ± 1.3	28.8 ± 3.6	31.2 ± 1.9	30.8 ± 2.6	30 ± 2.7	29.6 ± 1	30 ± 1.5	29.2 ± 2.3	33.2 ± 2.1	29 ± 1.7	32.5 ± 2.9	0.2
HR (bpm)	NEG	56 ± 1.8	62.5 ± 3	57.5 ± 2.8	60 ± 2.8	60 ± 4.9	59 ± 1.9	66 ± 4.2	61.5 ± 5.2	57.5 ± 3.6	56 ± 2.3	54.5 ± 2.2	56 ± 3.7	57.3 ± 2.1	59 ± 3.1	60 ± 2.2	0.10
	ATB	53.6 ± 3.5	57.6 ± 3.5	58.4 ± 5.3	56.8 ± 3.9	51.2 ± 2.3	51.6 ± 2.4	52 ± 2.2	56.4 ± 4.1	54.4 ± 2	53.6 ± 1	50.8 ± 1.2	50.8 ± 1.5	51.2 ± 0.8	46.7 ± 1	48 ± 0.8	0.25
	MSC	50.4 ± 0.4	54.8 ± 2.6	56.8 ± 1.9	55.2 ± 3.9	51.2 ± 2.7	50.4 ± 3.9	54.4 ± 3.2	53.6 ± 2.9	54.4 ± 2	51.6 ± 2.4	53.6 ± 0.7	53.6 ± 1.6	53,2 ± 2.4	52.5 ± 2.5	52.5 ± 1.5	0.32
RT (°C)	NEG	38.3 ± 0.1	38.2 ± 0.1	38.2 ± 0.1	38.3 ± 0.1	38.2 ± 0.1	38.1 ± 0.1	38.2 ± 0.2	38.4 ± 0.2	38.2 ± 0.2	35.5 ± 0.1	38.3 ± 0.1	38.2 ± 0.1	38.2 ± 0.2	38.2 ± 0.1	38.1 ± 0.2	0.14
	ATB	38.6 ± 0.5	38.0 ± 0.1	38.2 ± 0.2	38.7 ± 0.4	38.2 ± 0.2	38.4 ± 0.2	38.2 ± 0.3	38.3 ± 0.2	38.1 ± 0.3	38.1 ± 0.3	38.0 ± 0.3	38.3 ± 0.2	37.7 ± 0.3	37.8 ± 0.2	37.8 ± 0.2	0.18
	MSC	38.1 ± 0.1	38.0 ± 0.1	37.6 ± 0.2	38.0 ± 0.2	37.9 ± 0.2	37.7 ± 0.2	37.8 ± 0.2	37.6 ± 0.2	37.7 ± 0.3	37.9 ± 0.2	38.0 ± 0.1	37.6 ± 0.3	37.8 ± 0.3	38.1 ± 0.1	38 ± 0.1	0.9
MTD (°C)	NEG	0.46	0.63	0.96	−0.06	0,28	−0,65	0.19	0.025	0.53	2.33	1.04	1.21	0.39	0.2	0.35	0.12
	ATB	−0.17	0.65	0.43	0.51	−0.11	0.18	0.36	0.03	0.09	0.66	−0.05	0.18	0.26	0.5	0.6	0.14
	MSC	0.1	1.45	0.71	0.32	0.68	1.63	0.74	0.49	0.61	1.64	0.6	1.26	0.32	0.78	0.22	0.22
MY (lts)	NEG	17.7 ± 1.1	18.1 ± 1.8	18.0 ± 1.3	17.1 ± 1.4	17.6 ± 0.7	20.3 ± 2.6	13.5 ± 1.3	15.3 ± 2.4	15.9 ± 0.8	16.6 ± 2.4	19.0 ± 1	17.1 ± 1.6	17.8 ± 1.9	20.0 ± 1.8	16.3 ± 2	0.13
	ATB	17.9 ± 1.9	17.5 ± 2.2	15.7 ± 2.3	17.9 ± 1.8	17.5 ± 1	16.3 ± 2	15.8 ± 1.8	16.9 ± 1.4	16.0 ± 1.5	17.4 ± 1.6	16.3 ± 1.4	16.5 ± 1.6	16.5 ± 1.5	15.6 ± 1.8	16.8 ± 1.9	0.18
	MSC	16.8 ± 2.4	16.0 ± 2.5	15.8 ± 2.2	16.3 ± 2.2	14.3 ± 1.8	16.8 ± 2.3	17.2 ± 1.9	16.9 ± 1.3	16.7 ± 1.8	17.4 ± 1.7	19.2 ± 2.3	16.6 ± 1.9	15.6 ± 1.6	18.3 ± 2.2	17.8 ± 2.5	0.1

^a^RR = respiratory rate; HR = heart rate; RT = rectal temperature; MTD = mammary temperature difference; MY = milk yield. ^b^NEG and MSC groups were administered intramammarily on Days 4 and 10 with PBS and 2,5 × 10^7^ AT-MSC, respectively. ^c^ATB group was administered intramammarily on Days 4 and 5 with Pirlimycin.

Using a strip cup with dark surface test before each milking we found no traces of blood in milk; however, flakes or lumps were detected in milk from all cows at Day 2 post-challenge and intermittently until the end of the study. Average milk yield values were (range, max-min lts) 20.3 ± 2.6–13.5 ± 1.3 in NEG cows, 17.8 ± 2.5–15.6 ± 1.6 in ATB cows, and 19.2 ± 2.3–14.3 ± 1.8 in MSC cows (Table 3). Milk yield averages were no different (P < 0.05) between days post-infection or experimental groups. Significant changes in serum haptoglobin concentrations occurred over time for each challenge group (Table 4). Mean serum haptoglobin concentrations for NEG, ATB and MSC groups increased (P < 0.05) from day 1 (4.3 ± 0.4, 6.4 ± 4.4 and 4.6 ± 0.9, respectively) until day 2 (10.6 ± 0.4, 13.1 ± 5.9 and 13.3 ± 3.5, respectively) and day 5 (32.4 ± 11.4, 27.1 ± 2.3 and 24.2 ± 5.2, respectively); however, not significant treatment differences were observed. In comparison, serum amyloid A concentrations were not different (P > 0.05) between experimental days or treatments groups of cows.

**Table 4:** Haptoglobin and amyloid values in dairy cows with *S. aureus*-induced mastitis and treated with an intramammary suspension of AT-MSCs.

Item	Treat	Days post-infection								
		1	2	3	4^1,2^	5^2^	6	10^1^	15	P
Haptoglobin (µg/mL)	NEG	4.3 ± 0.4^a^	10.6 ± 0.4^b^	8.4 ± 4.1^b^	7.3 ± 4.1^b^	32.4 ± 11.4^c^	22.3 ± 8.4^c^	10.1 ± 5^b^	8.1 ± 4^b^	0.02
	ATB	6.4 ± 4.4^a^	13.1 ± 5.9^b^	12.4 ± 5.8^b^	9.8 ± 4.6^b^	27.1 ± 2.3^c^	9.8 ± 9.4^b^	11.1 ± 6.5^b^	10.4 ± 4.9^b^	0.04
	MSC	4.6 ± 0.9^a^	13.3 ± 3.5^b^	10.9 ± 5.5^b^	10 ± 5.2^b^	24.2 ± 5.2^c^	11.5 ± 6.4^b^	6.7 ± 2.3^a^	12.1 ± 4.5^b^	0.02
Amyloid A	NEG	3.3 ± 0.25	3.3 ± 0.25	2.7 ± 0.23	2.3 ± 0.47	2.5 ± 0.5	2.8 ± 0.47	3.6 ± 0.25	3.5 ± 0.29	0.19
	ATB	3.4 ± 0.4	3.2 ± 0.4	2.8 ± 0.6	2.3 ± 0.5	2.4 ± 0.43	2.8 ± 0.49	3.3 ± 0.5	3.5 ± 0.54	0.21
	MSC	3.6 ± 0.24	3.3 ± 0.42	2.8 ± 0.73	2.6 ± 0.6	3 ± 0.45	2.3 ± 0.51	1.9 ± 0	2 ± 0.24	0.32

^a^NEG and MSC groups were administered intramammarily on Days 4 and 10 with PBS and 2,5 × 10^7^ AT-MSCs, respectively. ^b^ATB group was administered intramammarily on Days 4 and 5 with Pirlimycin. ^a,b^Indicate significant (P < 0.05) difference among groups of cows. ^a-c^ Indicate significant (P < 0.05) difference between experimental days and treatments.

### Intramammary administration bovine fetal AT-MSCs in cows with mastitis caused by *S. aureus* resulted in lower bacterial count in milk compared to cows treated with vehicle

**Figure 3:**
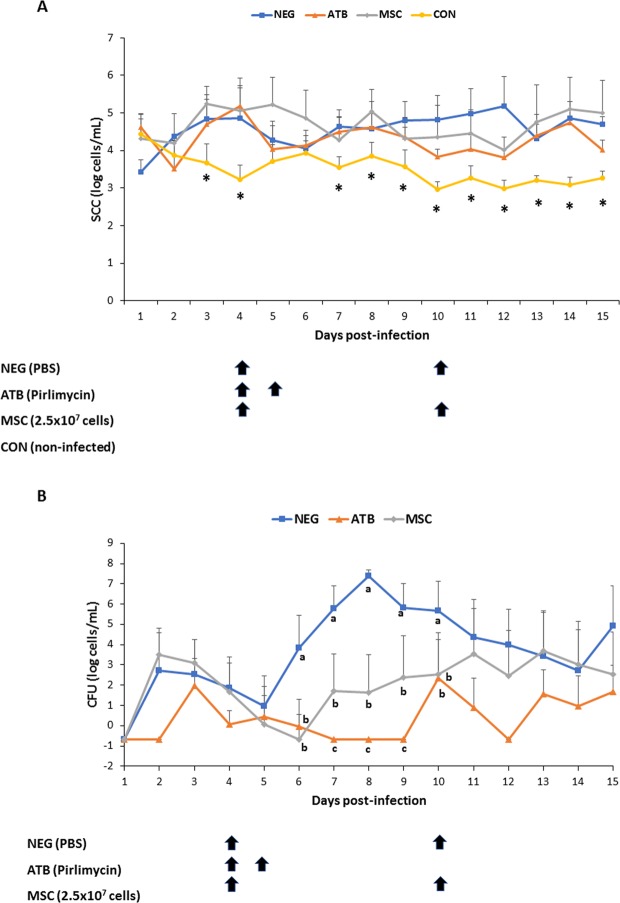
Somatic cell count (SCC) and colony forming units (CFU) in milk from mammary quarters with experimentally induced clinical mastitis by *S. aureus* and treated with bovine fetal AT-MSCs (MSC; n = 10 quarters), antibiotics (ATB; n = 10 quarters) or vehicle (NEG; n = 10 quarters). Non-infected mammary quarters (CON, n = 30 quarters) were used as negative controls. (**A**) Mean SCC log/mL from NEG, ATB and MSC infected mammary quarters were higher (Days 3, 4, 7, 8, 9, 10, 11, 12, 13, 14, and 15; P < 0.05) compared to CON mammary quarters. (**B**) Mean CFU log/mL in infected mammary quarters of MSC and ATB treatments were lower (Days 6, 7, 8, 9, and 10; P < 0.05) compared to NEG mammary quarters. Different superscripts (*, a,b,c) indicate significant differences between treatments. NEG, infected mammary quarters treated with vehicle on experimental days 4 and 10 (black arrows); ATB, infected mammary quarters treated with 50 mg of pirlimycin on experimental days 4 and 5 (black arrows); MSC, infected mammary quarters treated with a suspension of 2.5 × 10^7^ of AT-MSCs on experimental days 4 and 5 (black arrows); CON, non-infected mammary quarters inoculated with 1 mL of PBS on day 1 as a control.

From the day of the challenge and daily for 15 days, milk samples were collected from NEG, ATB and MSC mammary quarters to determine SCC and CFU numbers. Mean SCC log/mL from NEG, ATB and MSC infected mammary quarters were higher (Days 3, 4, 7, 8, 9, 10, 11, 12, 13, 14, and 15; P < 0.05) compared to CON un-infected quarters (Fig. 3). However, no differences were detected in mean SCC log/mL between treatment groups (P > 0.05) or sample periods (P > 0.05), and no significant interaction was noted between mean SCC log/mL of treatment groups and sample periods (P > 0.05). Mean CFU log/mL in infected quarters of NEG treatment were higher (Days 6, 7, 8, 9, and 10; P < 0.05) compared to values in mammary quarters of ATB and MSC treatments. Moreover, mean CFU log/mL in infected quarters treated with ATB were lower (Days 7, 8, 9; P < 0.05) compared to quarters treated with MSC. During the 15-day sample period, mean CFU log/mL in milk from infected quarters of NEG, ATB and MSC were (3.7 ± 1.5, 0.4 ± 0.7 and 2 ± 1.7, respectively). No significant interaction was detected between mean CFU log/mL of treatment groups and sample periods (P > 0.05). Bacteriological cure rates for NEG, ATB and MSC groups of cows were 0, 60 and 20%, respectively.

## Discussion

In the present study, we hypothesized that intramammary administration of AT-MSCs in cows with mastitis, would allow the delivery of MSC-derived bioactive factors at sites of lesion, where they may exert immunomodulatory control on the local immune response, strength the antimicrobial activity against mastitis pathogen and subsequently support the endogenous regenerative function of the damaged glandular tissue. This regenerative effect may reduce the therapeutic period for recovery of dairy cows and milk withhold, conforming an alternative or complement for the antibiotic therapy.

To our knowledge this is the first report that has evaluated the effect of the intramammary administration of MSCs for the treatment of bovine mastitis. Considering the absence of previous references and the lack of consensus on the appropriate dosage of MSCs for local therapy, the number of cells used in the present study (2,5 × 10^7^) was established according to previous reports in other large animal models including intra-articular administration in horses^21,22^, intra-uterine inoculation in mares^23,24^ and intra-myocardial injection in pigs^25^. After inoculation, MSCs exert their “homing” capacity by migrating to injured tissue, in response to chemotactic factors including stromal cell-derived factor-1 (SDF-1) and regulated on activation, normal T cell expressed and secreted (RANTES)^26^.

The use of allogenic MSCs is interesting in order to dispose of an “off-the-shelf” therapeutic product and avoid the period (4–6 weeks) associated with isolation and expansion of autologous MSCs. In this respect, abattoir-derived bovine fetal AT represent an abundant source for generating pools of AT-MSCs, allowing reduction in individual variations for therapeutic applications and offering the possibility to select the most suitable donors according to proliferation rate, differentiation ability or immunoregulatory properties. Nevertheless, failure of MSCs to activate immunosuppressive capacity may leave MSCs functioning much like antigen-presenting cells and able to promote inflammation *in vivo*^12^. In this scenario, the immunogenic recognition of MSCs may induce activation of T lymphocytes that trigger inflammatory response and cytolytic activity^27^. The helper (Th) (CD4+) and cytotoxic T (CD8+) lymphocytes may interact with alloantigens, which regulate the expression of selected molecules involved in the alloresponse^28^. This response is mediated by increased expression of interleukin 2 receptor (CD25), essential for the proliferation and differentiation of T lymphocytes^29^ and CD69, a marker of early activation that participates in the regulation of lymphocyte distribution^30^. In addition, the alloresponse downregulates the expression of L-Selectin (CD62L), which is involved in the adhesion of lymphocytes to peripheral lymph nodes^31^. Once the T lymphocytes are activated by the direct and indirect mechanisms, the release of various cytokines is generated, including IL-2, IFN-ɣ, and TNF-*ɑ*, and chemokines such as CCL2, CCL5 and CXCL3, that mediate an intense infiltration of macrophages and proliferation of T lymphocytes^28^.

In a safety trial, we found that administration of two repeated doses of allogenic AT-MSCs, separated by 10 days, did not induce changes in clinical and hematological variables in healthy cows during a 20-Day period. Moreover, we found no significant variation in gene expression profiles associated to PBL activation (CD4, CD8, CD25, CD62L and CD69) and proinflammatory cytokines (CCL2, CCL5, IL2, CXCL3, IFNγ, and TNFα). As it has been demonstrated in other species and diseases, application of repeated doses of MSCs is important in order to strength the therapeutic effect of MSCs^32,33^. However, recent reports have indicated that infusion of allogenic MSCs can induce immune memory in recipients^34,35^. Despite this, anti-donor response has been observed in some clinical trials, no major adverse events have been associated to administration of repeated doses of allogenic MSCs, which is considered a common therapeutic approach in commercial MSC-based therapies^36^. Overall, our data indicate that intramammary administration bovine fetal AT-MSCs in two doses separated by 10 days, did not induce local clinical effect in the mammary gland or activate systemic immune response in heifers.

In an efficacy trial, we found that quarters inoculated with *S. aureus* had higher SCC compared to non-infected quarters. Moreover, presence of flakes in milk with no swelling of the gland or systemic illness indicate that experimental inoculation of *S. aureus* resulted in mild clinical mastitis. This diminished inflammatory reaction, possible associated to reduced immune response, corresponds to the *in vivo* model recently described for induced mastitis after S aureus udder infection^37^. Among infected quarters, treatment with AT-MSCs resulted in similar SCC compared to treatment with antibiotics or vehicle. During inflammation, predominant types of SC are neutrophils followed by macrophages and lymphocytes, which enters the mammary gland from blood^38^. MSCs have been shown to induce immunomodulatory control on neutrophils, macrophages and lymphocytes by secretion of several factors including cytokines IL-6, IL-10, PGE2 and IDO^39–42^. We previously reported that bovine fetal AT-MSCs responds to inflammatory stimulation by up-regulation of IDO and IL-6^9^. This data generated under *in vitro* conditions suggested that AT-MSCs would be able to reduce SCC in quarters with mastitis. However, *in vivo* results from the present study indicate that AT-MSC intramammary inoculation in quarters with mastitis caused by *S. aureus* did not affect SCC in milk. *S aureus* has been reported to elicit a subtle immune reaction in the host after infection, through several mechanisms that include attenuation of NF-κB activation in mammary epithelial cells and TNFα, IL-6 and IL-8 in the mammary gland^43,44^. A subtle immune response in the mammary gland may have reduced the potential inflammatory activation of AT-MSC. Moreover, despite cows with intramammary infection by *S. aureus* increased haptoglobin levels over time, no significant differences were detected between treatment groups. Previously it has been reported that haptoglobin levels in milk and serum correspond with SCC, which support its use as a biomarker for mastitis detection^45,46^. Contrary to haptoglobin, amyloid A production is higher in the mammary tissue compared to the liver, which may explain the reduced sensitivity of the semi-quantitative test in serum and the lack of significant differences between treatment groups. Overall these results suggest that inflammatory conditions in the mammary gland were not able to induce secretion of immunomodulatory factors in AT-MSCs or secreted factors were insufficient to control inflammation mediated by migration of leukocytes from blood to milk and haptoglobin and amyloid A production in the mammary gland and liver.

Interestingly, in the efficacy trial, cows with mastitis caused by *S. aureus* and treated with bovine fetal AT-MSCs had lower CFU levels in milk compared to cows treated with vehicle. In a previous study, we reported that conditioned media from bovine fetal AT-MSCs has the capacity to reduce relative growth of mastitis causing *S. aureus* under *in vitro* conditions and that this antibacterial effect may be mediated by DEFβ1 and NK Lysin^11^. DEFβ1 is cysteine rich, cationic peptide, found in cow milk, that is capable of killing a broad spectrum of pathogens including *S. aureus*^47–49^. Moreover, a positive relationship has been described between SCC in milk and DEFβ1 gene expression, which was localized in epithelial cells of cow mastitic tissue^50^. NK Lysin is a cationic AP produced by cytotoxic T and NK cells that was isolated for the first time from porcine small intestine^51^. All four synthetic forms of NK-lysin peptides in the bovine had antibacterial capacity against *S. aureus*^52^. Despite levels of DEFβ1or NK Lysin were not analyzed in milk of cows treated with AT-MSCs, our results indicate that cells may exert an antibacterial effect against *S. aureus* in the mammary gland, which results in lower CFU in milk in MSC- compared to NEG-treated cows.

## Conclusions

Intramammary administration of repeated doses of allogenic bovine fetal AT-MSCs did not induce immune rejection or memory in healthy dairy heifers. Moreover, administration of this therapy to cows with mastitis caused by *S. aureus* resulted in lower bacterial count in milk compared to cows treated with vehicle. Overall, these results represent the foundation for the potential development of MSC-based therapy for the treatment of bovine mastitis; however, further studies are required in order to optimize the beneficial effects and determine the underlying mechanisms.

## Data Availability

All data generated or analyzed during this study are included in this article and the additional files.
